# CT Hounsfield units in assessing bone and soft tissue quality in the proximal femur: A systematic review focusing on osteonecrosis and total hip arthroplasty

**DOI:** 10.1371/journal.pone.0319907

**Published:** 2025-03-26

**Authors:** Tong-jie Yang, Peng-peng Wen, Xin Ye, Xiao-feng Wu, Cheng Zhang, Shi-yi Sun, Zi-xuan Wu, Guang-yi Zhang, Yi-fei Sun, Ren Ye, Cheng-kun Zhou, Hai-jun He

**Affiliations:** 1 Wangjing Hospital, China Academy of Chinese Medical Sciences, Chaoyang District, Beijing, China; 2 First Affiliated Hospital of Wenzhou Medical University, Wenzhou, Zhejiang Province, China; 3 The Third Affiliated Hospital of Zhejiang Chinese Medical University, Hangzhou, Zhejiang, China; 4 Zhejiang Chinese Medical University, Hangzhou, Zhejiang Province, China; 5 Beijing University of Chinese Medicine, Chaoyang District, Beijing, China; University of Perugia: Universita degli Studi di Perugia, ITALY

## Abstract

**Background:**

Computed tomography (CT) Hounsfield Units (HU) offer valuable insights into the changes in bone and soft tissue densities, playing a crucial role in the diagnosis and management of various proximal femur conditions. This systematic review aims to consolidate the application of HU in assessing tissue quality in the proximal femur, with a special focus on osteonecrosis of the femoral head (ONFH) and implications for total hip arthroplasty (THA), thereby addressing unresolved issues in these areas.

**Methods:**

We conducted a comprehensive literature search on MEDLINE/PubMed, EMBASE, Google Scholar, SpringerLink, Scops, Web of Science, and Bentham Science Publishers from inception to January 2024, following the PRISMA guidelines, to retrieve all studies relevant to the application of HU in assessing both bone and soft tissue quality of the proximal femur, particularly in the context of ONFH and THA. We systematically evaluated the key findings extracted from the included articles.

**Results:**

This systematic review included a total of 58 studies, involving 15,668 patients. The sample sizes ranged from 50 to 685, with the CT slice thickness varying from 0.5 mm to 10 mm. The results mainly focused on three areas: (1) the relationship between HU and the density of proximal femoral tissues (n = 33); (2) the assessment of HU in predicting the risk of femoral head collapse (n = 10); (3) the application of HU during the perioperative period of THA (n = 15).

**Conclusion:**

(1) HU can effectively contribute to the evaluation of bone and soft tissue densities in the proximal femur, and reflect local stress changes. (2) In ONFH patients, bone density does not decrease in the necrotic area of the femoral head before collapse. However, abnormally elevated HU at the outer boundary of the necrotic lesion are significant in assessing collapse risk. (3) HU can be used to preoperatively assess hip bone quality for THA, guide surgical approaches, predict intraoperative fractures, monitor postoperative bone ingrowth or absorption, identify and quantitatively evaluate periprosthetic loosening, and guide postoperative rehabilitation.

## 1. Introduction

Osteonecrosis of the femoral head (ONFH) poses a significant challenge in orthopedic practice, often leading to femoral head collapse and necessitating total hip arthroplasty (THA) in advanced stages [1,2]. The ability to accurately assess the quality of bone and soft tissue in the proximal femur is crucial for diagnosing ONFH, predicting femoral head collapse, and planning THA. Computed tomography (CT) Hounsfield Units (HU) have emerged as a valuable tool in this regard, offering insights into bone and soft tissue densities that are indicative of underlying pathological changes.

CT is a medical imaging technique that uses the absorption and attenuation of X-ray photons to produce cross-sectional images. Each tissue’s X-ray absorption is determined by its linear attenuation coefficient μ, with water typically set at 1. The CT value, expressed in Hounsfield Units (HU), represents tissue density [3]. Water has a CT value of 0 HU, air -1000 HU, and bone tissue typically ranges from 300 to 3000 HU. HU values are directly proportional to tissue density, with higher values indicating denser tissues [4].

Despite the potential of HU in clinical application, the literature presents a fragmented view, with studies focusing on various aspects of proximal femur assessment in isolation. This systematic review aims to consolidate current knowledge on the application of HU in the proximal femur, with a special emphasis on its role in ONFH and THA. By providing a comprehensive analysis of the available evidence, we seek to highlight the clinical utility of HU measurements, address unresolved issues, and suggest directions for future research.

## 2. Materials and methods

The authors adhered to the criteria outlined in the Preferred Reporting Items for Systematic Reviews and Meta-Analyses (PRISMA) guidelines for this review [5].

### 2.1. Search strategy

Two researchers (Cheng Zhang and Shiyi Sun) conducted a comprehensive literature search from inception to January 2024 on multiple databases including MEDLINE/PubMed (National Center for Biotechnology Information, NCBI), EMBASE(Ovid), Web of Science, Google Scholar, SpringerLink, Scops(Directscops), and Bentham Science Publishers. The search string included medical subject headings (MeSH) and free-text terms. By applying Boolean logic, the keywords used to select a maximum number of relevant studies were: (“Hounsfield Unit” or “HU” in subject terms) AND (“Osteonecrosis of Femoral Head” or “ONFH” or “Femoral Head Avascular Necrosis” or “FHAN” or “osteonecrosis” or “hip” or “coxa” in subject terms) AND (“microarchitectural” or “Collapse” or “subchondral” or “crescent sign” or “acetabular” or “Trabecular bones” or “bone marrow edema” or “double-line sign” or “sclerotic rim” in abstract). Additionally, a search was conducted in the reference lists of the selected articles and reviews to identify any potentially missed studies.

### 2.2. Inclusion and exclusion criteria

To guarantee quality and accuracy, only peer-reviewed journal papers with full-text availability were included. This study establishes the inclusion and exclusion criteria outlined in Table 1. Each paper is thoroughly reviewed to ascertain its eligibility for analysis.

**Table 1:** Inclusion and exclusion criteria.

Criteria	Description
Inclusion	Written in English
	Empirical studies
	All cited studies must have been approved by an ethics committee or an institutional review committee
Exclusion	Written in other languages
	Literature reviews, commentaries, or meta-analyses
	Studies that do not provide sufficient data on the topic or when the paper was not available.
	Observational studies, case series studies, and case reports were included.

### 2.3. Evidence quality assessment

**Fig 1:**
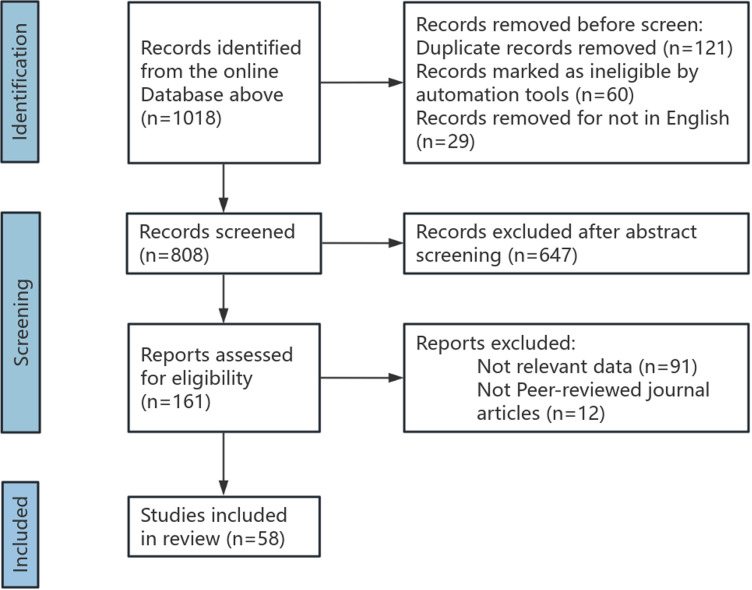
PRISMA flow chart of study selection.

Based on these criteria, the researchers conducted the PRISMA review process (Fig 1), which included identification, screening, eligibility assessment, and analysis. When discrepancies arose between the researchers, a third physician made the final decision regarding paper inclusion or exclusion. Additionally, the studies were evaluated using a modified Oxford Centre for Evidence-Based Medicine 2011, and each association was assigned a level of evidence [6] (S4 File). The assigned levels of evidence were discussed among team members until a consensus was reached.

To assess the robustness of the synthesized results, sensitivity analyses were conducted by excluding studies with the highest risk of bias. The results remained consistent, indicating that the overall findings are robust and not unduly influenced by any single study. Specific sensitivity analyses conducted include: Excluding studies with less than 50 patients; Removing studies that did not use standardized CT slice thickness; Analyzing the impact of excluding studies with the highest heterogeneity scores.

## 3. Results

### 3.1. Overview of findings

After several rounds of screening, 58 papers meeting the standard were eventually retained, involving 15,668 patients. The sample sizes ranged from 50 to 685, with the CT slice thickness varying from 0.5 mm to 10 mm. The results mainly focused on three areas: (1) the relationship between HU and the density of proximal femoral tissues (n = 33); (2) the assessment of HU in predicting the risk of femoral head collapse (n = 10); (3) the application of HU during the perioperative period of THA (n = 15).

### 3.2. HU can effectively detect the density of bone tissues in the proximal femur

Based on a literature review, we identified 15 studies demonstrating a strong correlation between HU and bone density in the proximal femur. These datas can help evaluate and quantify the bone quality of the proximal femur [7–21]. Ye [11] examined data from 680 patients who underwent CT and DEXA scans of the proximal femur between 2010 and 2020. HU was measured in four axial slices of the proximal femur, and Pearson correlation coefficients were utilized to compare these measurements with DEXA results. The study revealed a strong positive correlation between HU in the proximal femur and T-score, neck bone mineral density, and total hip bone mineral density (r =  0.777, r =  0.748, r =  0.746; all p <  0.001). The area under the curve for diagnosing osteoporosis using HU was 0.893 (p <  0.001), with a cutoff of 67 HU resulting in a sensitivity of 84%, specificity of 80%, positive predictive value of 92%, and negative predictive value of 65%. This trial not only confirmed the strong positive correlation between HU at the proximal femur and DEXA results, but also demonstrated the potential of HU for opportunistic screening of osteoporotic patients (other pertinent studies are presented in Table 2). Importantly, a study by Daniel [8] discovered that the correlation between HU and DEXA remained consistent across various age, sex, and scan interval groups when comparing patients with osteoporosis, osteopenia, and normal bone density. Based on the aforementioned studies, it can be concluded that HU obtained through CT can evaluate the bone density of the proximal femur.

**Table 2:** CT Hounsfield Unit evaluate the bone density of proximal femur.

Author/year	Positions	CT type		Layer thickness	Number of patients	Age of patients (yrs)	Evaluate conclusion	References
Gaoxiang Xu et al., 2023	Proximal femur		Siemens AG, Erlangen, Germany	Unkown	375; male/female: 135/240	Mean age: 77.81 ± 9.44	1. The CTh and HU values of specific cortex sites in the proximal femur were positively correlated with BMD of DXA at the hip.	[10]
							2. Thresholds for osteoporosis based on CTh and HU values could be utilized to screen osteoporosis and predict clinical outcomes.	
Kaifeng Ye et al., 2023	Proximal femur		Dual Source Computed Tomography DEFINITION, Siemens	5 mm	680; male/female: 165/515	Mean age: 63.66 ± 11.36	Proximal femur CT values had good positive correlation with DXA results, which could be used to opportunistic screening for potential osteoporosis patient.	[11]
Young-kyung Min et al., 2023	Femoral neck		Siemens (SOMATOM 128, Definition AS+) scanner (Siemens Healthcare, Forchheim, Germany)	3 mm	398; male/female: 156/242	Mean age: 58.35 ± 12.59	The modality using morphometric texture analysis with CT HU can be an additional diagnostic tool for osteoporosis and an alternative for DXA.	[12]
Sun-Young Park et al., 2022	From the femoral head to the lesser trochanter and the femoral neck		Using two MDCT scanners (SOMATOM Definition Edge, SOMATOM Definition Flash; Siemens Healthineers, Forchheim, Germany) in the standard single-energy CT mode.	1 mm	430; all female	Mean age: 65.4 ± 12.1	CT HUHA and mean-HU on 3D-VOI for predicting femoral osteoporosis showed similar diagnostic accuracy with better reproducibility of measurement, compared with 2D-ROI.	[9]
Mohamad Farhan Mohamad Amin et al., 2020	Pelvis (left hip)		Somatom Emotion 16 slices	Unkown	151; male/female: 9/142	17.9% age between 19–49; 31.8% age between 50–59; 29.8% age between 60–69; 16.6% age between 70–79; 4% age between 80–99	HU values derived from the hip, T7 and L3 provided a good to moderate correlation to t-scores with a good prediction for osteoporosis. The suggested optimal thresholds may be used in clinical settings after external validations are performed.	[13]
Fernando U Kay et al., 2021	Femoral necks		Biograph 64 PET/CT scanner (Siemens Healthineers, Erlangen, Germany)	5 mm	100; male/female: 66/34	Mean age: 53.94 ± 12.65	1. Increased age and low marrow SUVmax were associated with low BMDQCT at the lumbar spine (both P < 0.001), whereas increased age, female sex, and low marrow SUVmax were associated with low BMDCTXA at the femoral necks (P < 0.001, P < 0.001, P = 0.01, respectively).	[14]
							2. HU could be a simple and accurate approach for detecting patients at risk for osteoporosis-related fractures using PET/CTdata.	
Sindhura Bandaru et al., 2020	Hip		Opportunistic CT	Unkown	387; sex: unknown	Unkown	CT Hounsfield units (HU) measured bone mineral density (BMD) at the hip and spine can be used to diagnose osteoporosis.	[15]
N. Shankar et al., 2018	Femur bone, which consolidates neck, Ward’s triangle,trochanter district (more critical and lower), shaft cortex		Siemens Somatom, AS, Germany	0.5 mm	50; all female	Mean age of pre-menopausal (n = 18): 36.33 ± 8.7 Mean age of post-menopausal (n = 32): 55 ± 3	The Hounsfield is measured in neck of femur, trochanter head and shaft. Est volume is calculated through measured Hounsfield unit. The mean estimations of range and the volume of the proximal neck were diminished - 20% and 21% in the osteoporotic Indian ladies than in typical Indian ladies.	[21]
Daniel L Christensen et al., 2019	Proximal femur (comprised of femoral neck, trochanteric and intertrochanteric regions)		Unkown	1.5–3 mm	277; male/female: 41/236	Mean age: 63 ± 8	1. Quantifying bone mineral density (BMD) on CT using commercial software demonstrates good-to-excellent correlations with dual-energy x-ray absorptiometry (DEXA) results.	[8]
							2. An effective method of measuring HUs of the proximal femur from CT colonoscopy allows for opportunistic osteoporosis screening. These CT scans are frequently performed before initial DEXA scans are done and therefore may lead to earlier recognition of low BMD.	
							3. The linear regression model demonstrated that the overall positive correlation coefficient between HUs and the proximal femur T-score is not influenced by age, sex, or time between studies.	
David Donohue et al., 2018	Femoral head, femoral neck and intertrochanteric region		64-slice CT	Unkown	255: sex unknown	Unkown	1. CT imaging obtained for pelvic and acetabular fractures can identify patients with osteoporosis without additional radiation exposure or cost.	[16]
							2. The fitted ovoid region of interest is a standard feature in most CT scan platforms and is quite simple to perform.	
Seung-Ju Kim et al., 2017	Proximal femur		16-row multiple detector computed tomography (MDCT) scanner (MX8000; Philips Healthcare, Andover, MA)	Unkown	100; male/female: 20/80	Group I (25 hips): 82.3 (68.1 to 99.3); Group II (75 hips): 81.6 (65.1 to 97.9)	1. HU assessment using preoperative CT scan is associated with the presence of intra-operative fracture during bipolar hemiarthroplasty.	[17]
							2. We believe that HU values of the proximal femur could be used to assess local bone quality.	
Yee-Suk Kim et al., 2015	The head-neck junction of the femur, the middle portion of the femoral neck, and the intertrochanter of the femur		16-row-multiple detector computed tomography (MDCT) scanner (Somatom Sensation 16, Siemens Medical Solutions, Erlangen, Germany) or a 64-row-MDCT scanner (Brilliance 64-channel, Philips, Netherland)	3 mm	79; male/female: 23/56	mean age: 77.5 (range 56–93)	1. We found that a significant correlation between HU of pelvic dCT and BMD of DXA, and HU potentially provided an alternative method for determining regional BMD.	[18]
							2. Pelvic dCT could possibly be a supplementary method for initial diagnosis of osteoporosis and for initiation of treatment.	
Milka Marinova et al., 2015	Femoral neck		Philips AVE1, Brilliance 16, Brilliance 64 CT scanner	1–5 mm	234; male/female: 87/147	Mean age: 59.0 ± 13.2	1. Abdominal and particularly thoracic CT scans obtained for other clinical indications can sensibly be applied toward determining low BMD, detecting osteoporosis and identifying persons at increased fracture risk.	[19]
							2. Superiorly to DXA, fragility fractures can be found without additional imaging or radiation exposure which can initiate early adequate treatment.	
S. Y. Lee et al., 2015	Femoral head, femoral neck, greater trochanter		Light-speed VCT (GE Healthcare, Milwaukee, WI, USA)	1.25 mm	292; male/female: 80/212	Mean age: 73.2 ± 11.5	Lower extremity CT is a useful screening tool for osteoporosis, and peripheral bone attenuation on lower extremity CT adequately reflects central BMD on DEXA.	[7]
Shoji Baba et al., 2019	The superior one-third of the femoral head		Non-contrast CT images (scanner: Aquilion; Toshiba, Tochigi, Japan)	0.63 mm	101; male/female: 25/76	Mean age: 47.0 (range 20–83)	1. The assessment of HU values on CT was useful for the evaluation of BMD of the femoral head.	[20]
							2. The current assessment did not demonstrate reduced bone mineral density of the necrotic lesion in pre-collapse ONFH	

### 3.3. HU can effectively detect the density of soft tissues in the proximal femur

We conducted a literature search and included 18 studies that demonstrated the use of HU for assessing and quantitatively measuring tissue density and mechanical changes in the vicinity of the hip joint [22–38] (refer to Table 3). In a longitudinal study involving 473 individuals focusing on health and aging, Jung discovered a significant positive correlation between HU in thigh muscles and femoral neck bone density [26]. Lu indicated a significant correlation between the density of the overall hip joint bones and the density of the gluteus maximus (males: P =  0.012; females: P =  0.043) [38]. Thomas suggested that HU can be used to quantify the impact of resistance exercise on bone and muscle, including describing the spatial heterogeneity response of bone to different loads caused by muscle contractions [39]. The selected participants engaged in 16 weeks of resistance exercise. The first group (squat/deadlift, n =  7) performed 4 sets each of squats and deadlifts, while the second group (abduction/adduction, n =  8) performed 4 sets each of standing hip abduction and adduction exercises. Participants exercised three times a week, gradually increasing the load to reach maximum intensity. CT scans were conducted before and after training to evaluate the cortical bone density and volume, as well as muscle density and volume associated with each group. The results indicated that the squat/deadlift group experienced increases in both femoral neck cortical bone density and volume, as well as hip extensor cross-sectional area and HU. Similarly, the abduction/adduction group demonstrated increases in trochanteric cortical bone density and volume, along with hip adductor cross-sectional area and HU. Expanding on these findings, Fernandez [40] conducted further investigations by utilizing a specialized calculation formula that combined HU and hip muscle force. The study revealed that enhancing hip muscle strength could effectively reduce peak stresses experienced within the pelvis and acetabulum, with the rectus femoris, gluteus maximus, and iliac muscle playing crucial roles. Considering the research discussed earlier, HU measurements can effectively evaluate the density of soft tissues in the proximal femur.

**Table 3:** CT Hounsfield unit evaluate the soft tissue of proximal femur.

Author/year	Positions	CT type	Layer thickness	Number of patients	Age of patients (yrs)	Evaluate conclusion	References
Elisabeth et al., 2021	Hips, thighs, and calves	Spiral CT scanner (General Electric Medical Systems.London.UK).	2–5 mm	49: sex unknown	Mean age: 77 ± 5	1. Significant increases in Muscle cross-sectional area (CSA) were observed in 2 of the 8 muscles studied, namely the knee extensors (1.9%) and the hip adductors (2.8%). For RA, increases were observed in 4 of 8 muscle groups, namely the hip flexors (1.1 HU), hip adductors (0.9 HU), knee extensors (1.2 HU), and ankle dorsiflexors (0.8 HU).	[22]
						2. We conclude that this type of multicomponent physical activity program results in significant improvements in physical function despite relatively small changes in muscle size and quality of some, but not all, of the measured lower extremity muscles involved in locomotion.	
Hans et al., 2020	Hip, thigh and calf muscles	Piral CT scanner (General Electric Medical Systems)	5 mm	36; male/female: 21/19	Mean age: 77 ± 6	1. Muscle atrophy and fat infiltration, two indicators of deconditioning and weakness in elderly frail patients, are typically assessed by means of manual image analysis from computed tomography (CT) scans.	[23]
						2. Automated measurements were generally strongly correlated with manually encircled CSA in all muscle groups (R = 0.79-0.99, p < .05) and shortened the analysis time by 70% (p < .05). In m. iliopsoas, however, the CSA became overestimated (15%, p < .05) with thresholded measurements, while the assessment of both CSA and RA was underestimated in muscles with high-fat content (i.e., the gluteal muscles) and in individuals with high-fat infiltration.	
Ching-Di et al., 2018	From L4 to the proximal femur	GE Discovery CT750 HD lightspeed scanner	Unkown	91; male/female: 24/67	Mean age: 81.1 ± 12.2	In proximal femur fractures, elderly patients with sarcopenia are more likely to have prolonged hospitalization following surgery and require more blood transfusion volume during the perioperative period.	[24]
Thomas et al., 2010	Thigh muscle tissue	Memphis clinic site: Somatom Plus 4, Siemens, Erlangen, Germany, or PQ 2000S, Marconi Medical Systems, Cleveland, OH, USA; Pittsburgh clinic site: 9800 Advantage, General Electric, Milwaukee, WI, USA	10 mm	2833; male/female: 1370/1463	Between 70 to 79	1. In models adjusted by age, race, gender, body mass index, and percentage fat, decreased thigh muscle HU resulted in increased risk of hip fracture [RR/SD = 1.58;95% confidence interval (CI) 1.10-1.99], an association that continued to be significant after further adjustment for BMD.	[25]
						2. Decreased thigh muscle HU, a measure of fatty infiltration of muscle, is associated with increased risk of hip fracture and appears to account for the association between reduced muscle strength, physical performance, and muscle mass and risk of hip fracture. This characteristic captures a physical characteristic of muscle tissue that may have importance in hip fracture etiology.	
Jung Hee Kim et al., 2012	Between the pubic symphysis and the inferior condyle of the femur	SOMATOM Sensation 16; Siemens, Munich,Germany	Unkown	473; male/female: 242/231	Mean age of men: 73.6 ± 7.6; mean age of women: 72.4 ± 6.5	1. Leg muscle mass, knee extensor strength and thigh muscle HU values were significantly positively correlated with femoral neck BMD in both men and women. However, muscle strength was not a significant determinant for the presence of low bone mass after adjusting for muscle mass in multiple logistic regression analyses	[26]
						2. Decreased thigh muscle HU values, a measure of fatty infiltration of muscle, were independently associated with increased risk of low bone density in the elderly population.	
Youn-Jung Kim et al., 2021	The cross-sectional area of subcutaneous fat and skeletal muscle at the upper thigh level	128-Channel multi-detector CT scanner (Somatom Definition AS Plus; Siemens Healthineers, Erlangen, Germany)	3 mm	876; male/female: 230/646	Mean age: 79	The subcutaneous fat area (SFA) measured at the upper thigh level and 1 year mortality are positively associated in elderly patients with proximal femur fracture. SFA may be an independent prognostic biomarker for 1 year mortality of femur fracture.	[27]
Fujimoto et al., 2023	Thigh muscle tissue	Unkown	5 mm	44; male/female: 29/13	Mean age: 84.6 ± 7.0	1. The %LDM on the fractured side was higher in the thigh and erector spinae. The %NDM on the fractured side was lower in the thigh. There was no significant difference in the %IMAT for all muscles.	[28]
						2. The thigh on the fractured side showed asymmetry with low %NDM and high %LDM. This characteristic captures a characteristic of muscle tissue that may have importance in hip fracture etiology.	
Mustafa et al., 2023	The psoas, iliacus and gluteus medius muscles were evaluated at the disc of L5-S1 level	Unkown	3–5 mm	400; male/female: 138/262	Mean age: 78.49 ± 7.67	1. The mean HU values of the patients in the femoral neck fracture group were significantly higher than the intertrochanteric fracture group (p < 0.001, p = 0.008; respectively). At the same time, the mean HU values of the gluteus medius muscle were higher in the femoral neck fracture group (p < 0.001), but in contrast with the psoas muscle, the CSA values of gluteus medius muscle were significantly higher in the intertrochanteric fracture group (p = 0.017).	[29]
						2. Fatty degeneration of the psoas muscle among the muscles around the hip may affect the type of hip fracture. Elderly patients with strong psoas muscles may experience femoral neck fracture due to contraction and torsion during falling.	
Keong-Hwan et al., 2021	Muscle cross-sectional areas were measured in axial CT scan at the body level of the 4th lumbar vertebra (L4), intervertebral disc level between the 5th lumbar vertebra and the 1st sacral vertebra (L5-S1) and just below level of the lesser trochanter (LT).	Unkown	Unkown	44; male/female: 29/15	Mean age of Hip fracture group group: 79.4 ± 7.2; Mean age of Control group group: 79.4 ± 7.2	Poorer quantity and quality of psoas muscle and extensor muscles of the spine rather than whole body muscles may contribute to falls and were characteristic features of the hip fracture patients in this series. These findings should be considered when recommending a preventive exercise and rehabilitation protocol.	[30]
Ram et al., 2015	At the midthigh level	Philips Brilliance 64 CT scanner; Phillips Electronics N.V. Eindhoven, The Netherlands	10 mm	43; male/female: 23/20	Mean age: 79.9	After hip fracture, reduced muscle loading can result in muscle atrophy.The observed asymmetry is consistent with the effect of disuse and inflammation in the affected limb along with training effects in the unaffected limb due to the favoring of this leg with ambulation during the postfracture period.	[31]
Makoto et al., 2022	From the iliac crest to the femoral condyle	64-slice CT scanner (Optima CT660 Pro; GE Healthcare, Milwaukee, WI, USA)	2 mm	44; male/female: 1/43	Mean age: 65.5 ± 9.1	We found that fatty degeneration of the hamstrings, iliopsoas, and hip adductor muscles was significantly related to HRQoL in patients with hip osteoarthritis. These findings suggest that these muscles should be targeted during conservative rehabilitation for HOA and perioperative rehabilitation for THA.	[32]
Anton et al., 2007	In multiple hip and thigh muscle	Transaxial CT scans (General Electric Spiral scan; GE Medical Systems, London, UK)	10 mm	22; sex unknown	Mean age: 67	Major muscles functioning around the hip and knee showed substantial loss of strength and mass, which contributes to the reduced ambulatory capacity of OA patients. Reduced muscle CSA could not fully explain the loss in strength. Infiltration with fat or other non-contractile components, as indicated by a reduced RD, in OA limb muscles was substantial.	[33]
Taku et al., 2021	Gluteus maximus (G-max), gluteus medius (G-med), tensor fasciae latae, internal obturator muscle, and external obturator muscle	Siemens, Germany	0.6 mm	64; male/female: 12/52	Mean age of PL group: 67.1 ± 9.9 ; Mean age of AL group: 67.4 ± 10	The PL approach can lead to degeneration of the internal and external obturator. The AL approach is more beneficial for recovering the G-med, tensor fasciae latae, and internal obturator muscle than the PL approach.	[34]
Michael et al., 2018	Psoas major, gluteus medius and minimus muscles	Unkown	3 mm	28; male/female: 11/17	Mean age: 54.4 ± 14.8	Age and height, as well as CT-derived visceral-to-subcutaneous fat area ratio and waist circumference significantly correlate with postsurgical HHS scores following THA. Our study suggests that parameters derived from cross-sectional CT imaging can be useful additional preoperative planning tool for THA.	[35]
A Rasch et al., 2009	Muscles of the hip, thigh, calf and back	Transaxial CT (General Electric Spiral scan; GE Medical System, London, United Kingdom)	Unkown	22; male/female: 4/18	Mean age: 67 (54 to 77)	There was persistent muscle atrophy in muscles acting about the hip two years after THR. We suggest that an earlier operation or a more intensive rehabilitation may reverse these changes.	[36]
Xiangjun Hu et al., 2021	From the center of the acetabulum to the center of the femoral head in the acetabular coordinate system	SOMATOM Definition AS1; Siemens, Germany	0.35 mm	18; male/female: 13/5	Mean age: 60.6 ± 9.0	An increase of 2–3 mm in FO could improve the abductor and external rotator function following a THA. Accurate surgical planning with optimal FO reconstruction is essential to restoring normal hip muscle function in THA patients.	[37]
Lu Yin et al., 2020	Gluteus maximus muscle (G.MaxM), trunk muscle at the vertebrae L2 level, and mid-thigh muscle	Toshiba Aquilion CT scanner (Toshiba Medical Systems Division, Tokyo, Japan).	10 mm	301; male/female: 194/107	Mean age: 68.4 ± 6.1	We observed positive associations of the gluteus and thigh muscle size with proximal femur volumetric BMD. Specifically, the gluteus maximus muscle CSA was associated with trochanter cortical vBMD in both men and women.	[38]
						2. The current assessment did not demonstrate reduced bone mineral density of the necrotic lesion in pre-collapse ONFH	
Thomas et al., 2014	The proximal femora(between the superior aspect of the acetabulum and the inferior aspect of the lesser trochanter)	Unkown	2.5 mm	22; male/female: 9/13	Mean age: 39.65	1. VBM showed different effects of ABADD and SQDL exercise, with the former causing focal changes of trochanteric cortical bone, and the latter showing diffuse changes in the femoral neck and head.	[39]
						2. ABADD exercise increased adductor CSA and HU, whereas SQDL exercise increased the hip extensor CSA and HU.	

## 4. Discussion

### 4.1. Advantages of HU in assessing proximal femur bone density compared to dual-energy X-ray absorptiometry (DEXA)

While DEXA is widely recognized as the “gold standard” for assessing bone quality [41], its application in ONFH patients presents challenges. Due to structural changes like trabecular loss, femoral head collapse, and increased density at certain regions, DEXA often struggles to accurately assess bone density in the proximal femur [8,42]. In response, multiple studies have explored HU measurements as an alternative for assessing bone quality in this region, demonstrating several distinct advantages over DEXA.

Firstly, HU offers a broad application scope, with quantitative bone density assessments that do not require additional radiation or costs. In addition to hip-specific CT scans, HU measurements can also be obtained from existing abdominal [8] or pelvic CT images [ 16,18], making it easier to identify high-risk patients with localized osteoporosis. This accessibility facilitates early intervention, helping to reduce the risk of fragility fractures and associated conditions.

Secondly, HU enables enhanced visualization of bone microstructure. Unlike DEXA, which cannot distinguish between cortical and trabecular bone [43], HU can separately quantify these components. For instance, studies by Lim [44] demonstrated a positive correlation between certain HU measurements in trabecular regions and osteoporosis presence, while specific cortical HU values correlated negatively with osteoporosis. These findings illustrate HU’s ability to offer insights into osteoporosis progression based on distinct cortical and trabecular characteristics.

Lastly, HU minimizes interference from overlapping tissues, a common issue with DEXA. DEXA measurements can be affected by overlapping tissues such as muscle, fat, and water, potentially leading to inaccurate bone density readings [7]. For example, subclinical femoral head collapse can cause bone marrow edema, reducing radiographic density and potentially masking the actual bone condition on DEXA scans [45]. In obese patients, excess fat around the hip and abdomen further complicates DEXA readings, as adipose tissue absorbs X-ray energy, resulting in lower apparent bone density. Conversely, CT scans using HU can directly measure the density of trabecular bone layers, accurately distinguishing bone from surrounding tissues and providing a clearer, interference-free assessment [9].

### 4.2. HU guiding insights into proximal femur bone density and its mechanical implications

HU is closely associated with the mechanical strength of local tissue. The distribution of subchondral bone density, as indicated by HU, can indirectly infer the distribution of stress and the joint’s loading history [46]. Initial studies combining HU-based bone density with indentation tests revealed that high HU values strongly correlate with increased mechanical strength, while low HU values indicate lower strength, with density concentrated primarily in the medial and central regions of the proximal femur [47]. Other studies reported strong correlations, with determination coefficients (R²) from 0.74 to 0.97 and Pearson coefficients between 0.86 and 0.98, when comparing density with mechanical strength across various sites [48]. Hoechel’s findings [49] further confirmed that high-density regions, such as the anterior superior acetabulum and femoral head articular surface, align with areas of high mechanical strength, although individual variation exists. While quantitative comparisons between bone density and mechanical strength are still developing, these findings support HU’s utility in inferring localized stress changes within bone tissue.

### 4.3. HU reveals contrasts between fat and muscle in the proximal femur

In addition to analyzing and quantifying muscle tissue composition, HU can precisely distinguish between fat and muscle by utilizing specific attenuation coefficients for each tissue type. This capability enables the description of various physiological and pathological conditions. Anton’s study [33] demonstrated that a 10% increase in tissue fat decreases HU by 0.75-1, with higher fat levels in gluteal muscles indicating significant muscle wasting and reduced mobility in hip osteoarthritis patients. In-depth research by Mustafa [28] revealed that fatty degeneration of the lumbar muscles can influence the type of hip fracture. Elderly patients with robust lumbar muscles may experience femoral neck fractures due to contraction and twisting during falls. Overall, HU effectively identifies ectopic fat in skeletal muscles, linking increased fat deposition to impaired muscle function and metabolic health. This makes HU a valuable tool for assessing soft tissue around the hip [27], supporting early intervention and non-invasive treatment strategies [40].

### 4.4. The application of HU in the evaluation of ONFH

Femoral head collapse is a critical event in the progression of ONFH, impacting the hip joint’s outcome. However, ONFH is often asymptomatic prior to collapse, making early diagnosis and timely intervention challenging. Current consensus suggests that collapse risk depends on factors like necrotic lesion size, location, and stress distribution, emphasizing the need to assess collapse risk for appropriate treatment planning [50].

Currently, there are varying opinions on changes in density within the pre-collapse area of osteonecrosis. Lulu suggested that bone resorption in the necrotic area lowers density, weakens mechanical strength, and serves as an early indicator of collapse risk [51]. However, previous studies have not demonstrated a decrease in bone density within the necrotic lesion. Notably, prior evaluations using DEXA may have introduced inaccuracies due to overlapping images from the acetabular wall and femoral head. To address this, several researchers have employed HU for a more precise assessment of femoral head density and bone microstructure, offering clearer insights into actual bone density conditions within necrotic regions.

Consistently, multiple studies have demonstrated that bone density in pre-collapse necrotic lesions remains unchanged [20,52,53]. For instance, Shoji [20] used HU to assess bone density in pre-collapse ONFH patients compared to controls, selecting the upper one-third of the femoral head as the region of interest (ROI). The study, using propensity score matching, found no significant difference in HU values between ONFH patients and controls within this ROI. Similarly, Cheng’s research revealed no significant differences in bone density or micro-mechanical properties between the necrotic and healthy trabecular areas [52]. This study also conducted histopathological and immunohistochemical analyses, finding increased osteoclast activity in the subchondral bone and necrotic areas, alongside increased osteoblast activity in the sclerotic region, indicating a reduction in macroscopic mechanical strength associated with shifts in osteoblast and osteoclast activity. Kazuyuki’s study corroborated these findings, observing heightened osteoclast activity within the necrotic femoral head [53]. These results suggest that HU values within the necrotic lesion remain stable until structural disruptions such as trabecular rupture, bone resorption, or bone marrow edema occur, leading to collapse. Thus, HU does not typically decrease in the pre-collapse stage.

Given that the HU of the necrotic lesion does not decrease, the question arises: How can we predict femoral head necrosis? Numerous studies have indicated that the outer boundary of the necrotic lesion plays a pivotal role in assessing the risk of collapse [20,54–58].

In ONFH, a demarcation line separates necrotic from viable tissue, marking the onset of pathological phases including ischemia, repair, and sclerosis. Baba’s study [20] revealed that ONFH patients who experienced femoral head collapse had significantly higher HU values at the lesion’s outer boundary compared to non-collapse cases, though there was no significant HU difference in the anterior upper part of the femoral head between groups. Histological studies of femoral head specimens consistently link collapse with subchondral fractures at the lesion’s boundary [54]. Finite element analysis further supports this, showing stress concentration at the outer border of the femoral head prior to collapse, coinciding with alterations in the sclerotic margin [55]. According to Li’s research data [56], the variation in local stress distribution is responsible for the HU changes at the lateral border before the occurrence of femoral head collapse. In a retrospective study of 40 ARCO stage II ONFH patients, the mean maximum von Mises stress levels were significantly higher in the collapse group (2.955 ± 0.540 MPa) than in the non-collapse group (1.923 ± 0.793 MPa) (P <  0.01). Building on these findings, Kubo reported that collapse rates at the sclerotic border in the acetabulum’s weight-bearing region varied significantly across different sections, with the highest rate (81%) in the outer third [57]. These findings indicate that stress is concentrated at the outer border, leading to shear stress between the thickened repair zone and adjacent necrotic trabeculae, resulting in subchondral fractures [55,58]. Note that HU can also differentiate stress-induced femoral head insufficiency fracture (SIF) from ONFH. Kawano’s research [59] reported significantly lower bone volume fraction, trabecular thickness, and bone density in collapsed areas of ONFH compared to adjacent non-collapse regions, whereas SIF cases showed no such microstructural differences between collapsed and non-collapsed areas, highlighting distinct pathogenic mechanisms between the two conditions.

In conclusion, detecting HU changes at the outer boundary of necrotic lesions offers valuable insights into collapse risk in ONFH patients, supporting timely evaluation and intervention to potentially alter the disease course.

### 4.5. Application of HU in perioperative THA

HU provides localized insights into bone and soft tissue quality around the hip, making it valuable for preoperative assessment, surgical planning, and prognosis in THA patients.

#### 4.5.1. Application of HU in preoperative assessment for THA.

Patients undergoing orthopedic surgery often experience poor bone health, with over 50% of joint replacement patients presenting with osteoporosis or osteopenia [60]. A cross-sectional study found that 26% of THA patients had osteoporosis [61], yet fewer than 4% of surgeons routinely measure bone density [60]. Even when osteoporosis is diagnosed, targeted pharmacological interventions are rarely used [62], despite evidence linking poor skeletal health with negative recovery outcomes, higher complication rates, and increased revision surgeries [63, 64]. Utilizing HU for osteoporosis diagnosis enables effective intervention, reducing complications and revision rates [65], and preserving bone mass post-surgery [66]. Additionally, HU’s adaptability allows measurements from any targeted region, enhancing its utility in clinical practice.

#### 4.5.2. Surgical approach guidance and intraoperative fracture prediction in THA using HU.

HU measurements offer valuable guidance for optimizing surgical approaches and predicting fracture risks in THA. A retrospective study involving 64 patients found that the lateral approach significantly reduced HU values in obturator muscles, indicating potential muscle degeneration, whereas the anterior-lateral approach increased HU in gluteus medius and tensor fasciae latae muscles, suggesting better muscle preservation [34]. Noda’s comparison of the “Bald Spot” technique with conventional trochanteric nail insertion further supports the role of HU in surgical planning, showing that the BS technique minimizes damage to the gluteus medius [67],

Additionally, Boomsma’s study of 317 THA patients found that HU values above the acetabulum were significantly lower on the operated side [68], highlighting changes in bone density that could signal the need for revision surgery [69]. Building on this, Nishi’s study of 301 patients linked lower preoperative HU levels in specific acetabular regions to a higher intraoperative fracture risk, with fractures most common in the superior aspect of the acetabulum (40%) [70]. These findings demonstrate that HU effectively predicts fracture risk in “weaker” regions, underscoring the importance of preoperative HU assessment in enhancing surgical precision and patient safety.

#### 4.5.3. Application of HU in the assessment of postoperative THA.

HU effectively assesses changes in bone and muscle density following THA. In a 12-year retrospective study of 11 THA patients, Lengsfeld observed a 50-150 HU decrease (10%) in femoral density within the first year post-surgery, reaching up to 400 HU (30%) after 12 years, with the most significant density loss near the distal lesser trochanter [71]. Gislason’s findings supported this trend [72]. Additionally, research has shown sustained muscle atrophy up to two years post-THA, with reductions in radiodensity of key hip muscles, including the gluteus maximus (10.1 HU) and gluteus medius (5.6 HU), compared to the healthy limb [36]. These insights support the need for targeted postoperative rehabilitation.

HU also enables monitoring of bone remodeling and implant stability. In animal studies, Miori [73] found a strong correlation between HU values and implant stability metrics—such as insertion and removal torque values—indicating HU’s potential for quantifying primary implant stability and identifying peri-implant loosening. Retrospective studies by Jixing Fan further demonstrated a significant correlation between lower femoral head HU values and higher prosthesis failure rates, with failure group HU values significantly lower than non-failure group values (133.25 ±  34.10 vs. 166.12 ±  42.68, p = 0.004) [74]. These findings highlight HU’s role in specific monitoring of bone remodeling and implant success.

## 4.6. Advantages and shortcomings

From a technical standpoint, HU is a relative value obtained through attenuation coefficients calibrated against water, rather than absolute value. Consequently, variations in CT scanner parameters, including slice thickness, brands, models, algorithms, or detection conditions can lead to differences in the data generated by HU, resulting in a lack of repeatability in image measurement data [75–77]. Calibration of CT scanners, ensuring standardized imaging protocols and calibration procedures, is essential to mitigate these discrepancies. Standardizing scanning parameters across different devices and protocols would enhance the reproducibility and consistency of HU measurements. Future research should prioritize not only standardizing these parameters but also establishing a universal calibration system for HU measurements, reducing variability and improving diagnostic reliability. Consequently, HU can only serve as a supplementary tool for diagnosing osteoporosis in conjunction with DEXA. Future research should aim to establish a more comprehensive and standardized evaluation system based on HU, which would enhance the guidance for diagnosing and treating patients with ONFH.

Regarding specific operations, because three-dimensional ROI selection cannot encompass the entire necrotic lesion of the femoral head and due to variations in trabecular bone density, errors may occur in HU depending on the position and size of the ROI. This can hinder the accurate reflection of the likelihood of collapse in extensive necrotic lesions [78]. Further research is needed to improve the accuracy of these measurements.

Regarding the methodological quality of the included studies, we recognize significant heterogeneity due to variations in study design, population characteristics, outcome measures, and the observational and retrospective nature of most studies, affected by differences in race, gender, age, and socioeconomic patterns. This diversity, compounded by moderate to low levels of evidence, selection bias, and limited sample sizes, may challenge the generalizability of our findings. Future research must prioritize standardized approaches, employing rigorous frameworks, and incorporating larger and more diverse sample sizes as well as prospective designs. A critical appraisal of study quality using standardized tools will aid in identifying and addressing potential biases, including publication bias. This rigorous evaluation is essential for enhancing the review’s robustness and advancing our understanding of HU’s role in assessing osteonecrosis of the femoral head (ONFH), ensuring findings are more reliably applied in clinical contexts.

## 5. Conclusions

The systematic review demonstrates that Hounsfield Units (HU) can effectively assess the density of bone and soft tissue in the proximal femur, reflecting local stress changes. Additionally, bone density in the necrotic area of the femoral head of ONFH patients does not decrease before collapse, and abnormally elevated HU at the outer boundary of the necrotic lesion is significant in assessing the risk of collapse. HU can also evaluate hip bone quality prior to THA, predict intraoperative fracture, and monitor postoperative bone growth or absorption. Furthermore, HU can identify and quantify periprosthetic loosening, guide surgical approaches and postoperative rehabilitation. In conclusion, despite limitations, CT Hounsfield Units remain a valuable tool for evaluating osteonecrosis of the femoral head and deserve further investigation and promotion.

## Supporting information

S1 File PRISMA checklist. (DOCX)

S2 File Included studies. (XLSX)

S3 File Inclusion and exclusion table. (XLSX)

S4 File Appendix A. (DOCX)
