# Supplementary material for: CT Hounsfield units in assessing bone and soft tissue quality in the proximal femur: A systematic review focusing on osteonecrosis and total hip arthroplasty
Source: PLoS One. 2025 Mar 26;20(3):e0319907. doi: 10.1371/journal.pone.0319907 (PMC11940759; doi:10.1371/journal.pone.0319907)
Supplement: S4 File — (DOCX) [file pone.0319907.s004.docx]

Appendix A .Level of evidence for rating studies and grading recommendations.

| **Level** | **Type of Evidence** |
| --- | --- |
| 1a | Systematic review with homogeneity of randomized controlled trials |
| 1b | Individual randomized control trial with a narrow confidence interval |
| 1c | All-or-none related outcome |
| 2a | Systematic review with homogeneity of cohort studies |
| 2b | Individual cohort studies and low-quality randomized clinical trials |
| 2c | Ecological studies |
| 3a | Systematic review of case-control or retrospective studies |
| 3b | Individual case-control or retrospective studies |
| 4 | Case series and case reports |
| 5 | Expert opinion |
| **Grades of Recommendation** | |
| A | Consistent level 1 studies |
| B | Consistent level 2 or 3 studies or extrapolation from level 1 studies |
| C | Level 4 studies or extrapolation from level 2 or 3 studies |
| D | Level 5 evidence or contrasting results reported among studies |
